# Building Blocks of Global Health Mentorship: Motivation, Expectations, and Institutional Support

**DOI:** 10.5334/aogh.1537

**Published:** 2019-03-18

**Authors:** Karen Charron, Anna Kalbarczyk, Nina A. Martin, Emily A. Combs, Marie Ward, Elli Leontsini

**Affiliations:** 1 Department of International Health, Johns Hopkins Bloomberg School of Public Health, US; 2 Johns Hopkins Center for Global Health, US; 3 Institute for Health Metrics and Evaluation, University of Washington, US

## Abstract

**Background:**

Global health education and training experiences are in high demand. Mentorship plays an important role in successful training, but academic institutions often lack formalized mentorship support. This study aimed to evaluate perceptions of global health mentorship across disciplines at Johns Hopkins University and to understand how to better support faculty mentorship for global health training.

**Methods:**

This is a retrospective study that used qualitative methods to assess the perceptions of students who participated in the Johns Hopkins Center for Global Health (CGH) field placement program from 2011–2013 and CGH faculty who may have served as their mentors. Qualitative data was gathered through 30 individual in-depth interviews and 4 focus groups capturing both faculty and student perspectives. Data were analyzed inductively until thematic saturation was reached; a theoretical model, which we call the “building blocks of global health mentorship” model, emerged to serve as an analytical and synthesizing framework.

**Findings:**

A series of factors influenced global health mentorship from an individual to institutional level, including motivation, expectation alignment, finances, time, and knowledge. Both students and faculty reported the importance of motivation and aligned expectations to the mentorship experience and, more broadly, the overseas experience. Mentorship relationships were identified by students and faculty as either a catalyst or a hindrance to the training experience from both a personal and a professional point of view. Many faculty mentioned insufficient institutional support and financial resources, which negatively influenced their capacity to serve as mentors.

**Conclusions:**

Many factors, ranging from individual to institutional, influence mentorship for both faculty and students, which in turn influence international experiences. The underlying role of institutional support emerged as a highly salient influencing factor. Global health programs should harness the faculty and students’ motivations and expectations, as well as provide better support to faculty serving as mentors.

## Introduction

Demand for global health education and training programs has increased rapidly over the past 20 years. Academic institutions have responded by developing centers and institutes, interest groups, and degree programs ranging from the undergraduate to doctoral level. The number of global health programs offered in North America grew tenfold between 2001 to 2011, and now, more than 250 North American institutions have global health educational offerings [1]. In 2009, the Center for Strategic and International Studies (CSIS) surveyed 52 universities with global health programs to better understand their offerings and focus [2]. Of all university-based activities, the priority was the delivery of student experiences. In fact, 81% reported that at least one quarter of their activities focused on education, training, and mentorship [2].

Global health field training has been shown to provide beneficial skills and improve cultural competency [3,4,5]. When exposed to global health training, medical students are more likely to enter primary care medicine, receive public health degrees, and work within poor and ethnic minority populations [6]. In addition to medical students, global health field training is available at many institutions for public health, nursing, engineering, business, and students in other disciplines [1,2]. An evaluation of a National Cancer Institute-funded traineeship for cancer epidemiology graduate students selected to participate in a summer global health training program demonstrated that mentorship played a role in positive short- and long-term student outcomes as measured by student publications of their field work and employment in the field after three years [7]. It is reasonable to expect that establishing a model of considerations in a mentor-mentee relationship within the global health curriculum would not only improve student experience, knowledge, and skills, but also result in a higher likelihood of continued international engagement from the student and stronger relationships with colleagues and communities overseas.

Mentorship has not been clearly defined. A literature review of academic mentorship by Jacobi et al. found that there is no universal definition or outcome measures for mentorship [8]. Berk et al. propose “a false sense of consensus exists because at a superficial level, everyone ‘knows’ what mentorship is. However, upon closer examination, there is a wide variation in operational definitions” [9]. The National Academy of Sciences says that mentoring involves “informal, individual to individual, relationships based on nothing less than reciprocated trust, respect, understanding and even empathy” [10]. Johnson, in his chapter “A framework for conceptualizing competence to mentor,” distinguished mentoring from advising: “In contrast to other faculty roles, mentoring requires a faculty member to engage in a dynamic, emotionally connected, and reciprocal relationship with the protégé” [11].

One of many challenges to mentorship in global health may be the remote nature of field experiences for extended periods of time. There is some research on clinical mentors in global health that has shown the critical role mentors play in both good and bad experiences [12]. Shah et al. argue “mentors need to anticipate the unique concerns of each trainee” [12]. Toolkits have been developed and modified for mentors and mentees conducting clinical international electives [13]. The Standing Committee on Postgraduate Medical and Dental Education (SCOPME) published a report in 1998 stating “an experienced highly regarded person (the mentor) guides another individual (the trainee) in the development and re-examination of his or her own ideas, learning, personal, and professional development” [14]. Very little is known about the challenges of interactions with mentors from non-clinical fields (i.e., public health, engineering, business, etc.).

In this paper, based on the aforementioned operational definitions and guidelines, mentorship is defined as a partnership in personal and professional growth and development. This study was designed to evaluate student and faculty perceptions of global health mentorship and to understand how to improve mentorship experiences for both mentors and mentees.

## Methods

**Study Design.** This is a retrospective study that took place from 2013 to 2015 and used a qualitative research design to explore factors affecting mentorship in international student learning experiences.

**Study Population.** Student and faculty were recruited from the Johns Hopkins University Center for Global Health (CGH), which was founded in 2006 to address the increasing demand for global health opportunities among students and faculty across disciplines. More than 430 university faculty were affiliated with the CGH, and approximately 120 of those affiliates have worked with students through the Center’s training programs. Many of these faculty affiliates have served as mentors to their junior faculty and to students in non-CGH projects. One of the Center’s largest training programs, the Global Health Established Field Placements (GHEFP), provides $3,500 travel grants to students to work with faculty members on their research or to practice projects overseas.

**Sampling Strategy.** The sampling frame included all 431 CGH-affiliated faculty members from the Johns Hopkins University who had served as global health mentors during their tenure with the university and the 186 undergraduate and graduate students who participated in the GHEFP from 2011 to 2013. All training program participants and CGH-affiliated faculty were eligible to participate in the study. A convenience sample of 8 students and 20 faculty were selected for interviews based on their availability and the availability of the interviewers.

**Data collection methods.** In 2014 and 2015, two teams of independent graduate student research groups collected qualitative data as part of a two-series graduate-level qualitative research and analysis course at the Johns Hopkins Bloomberg School of Public Health. Training-program-specific data were collected from January to April 2014. The mentorship theme was established in the following year, upon which a second round of data was collected by a new student team between January and April 2015.

In-depth interviews were conducted with 8 students and 20 mentors for a total of 28 interviews. Recruitment was conducted through email invitations, and interested potential participants were contacted to schedule an interview. Focus groups were conducted with students and faculty separately to gather a broader community perspective on field experiences and mentorship. Four focus groups were conducted in total: 1 student focus group had 5 participants, and 3 faculty focus groups had 14 total participants (5, 6, and 3 participants in each group). Topics covered in focus group discussions were similar to those in interviews. All interviews and focus groups were conducted in English, audio-recorded by digital recorders, and transcribed.

**Conceptual Framework.** In the previous study on factors influencing the student global health experience, a socio-ecological (SE) model served as a conceptual framework in which mentorship played a cross-cutting role [15]. Mentorship was identified as an interpersonal factor that spanned the continuum of the students’ global health experience, from pre-departure and preparation through the practicum to pre-departure. This research expands on the concept of mentorship within the SE model and identifies factors that influence mentorship across the SE levels (individual, interpersonal, institutional, and societal), which we are calling the Building Blocks of Global Health Mentorship Model.

**Figure 1:**
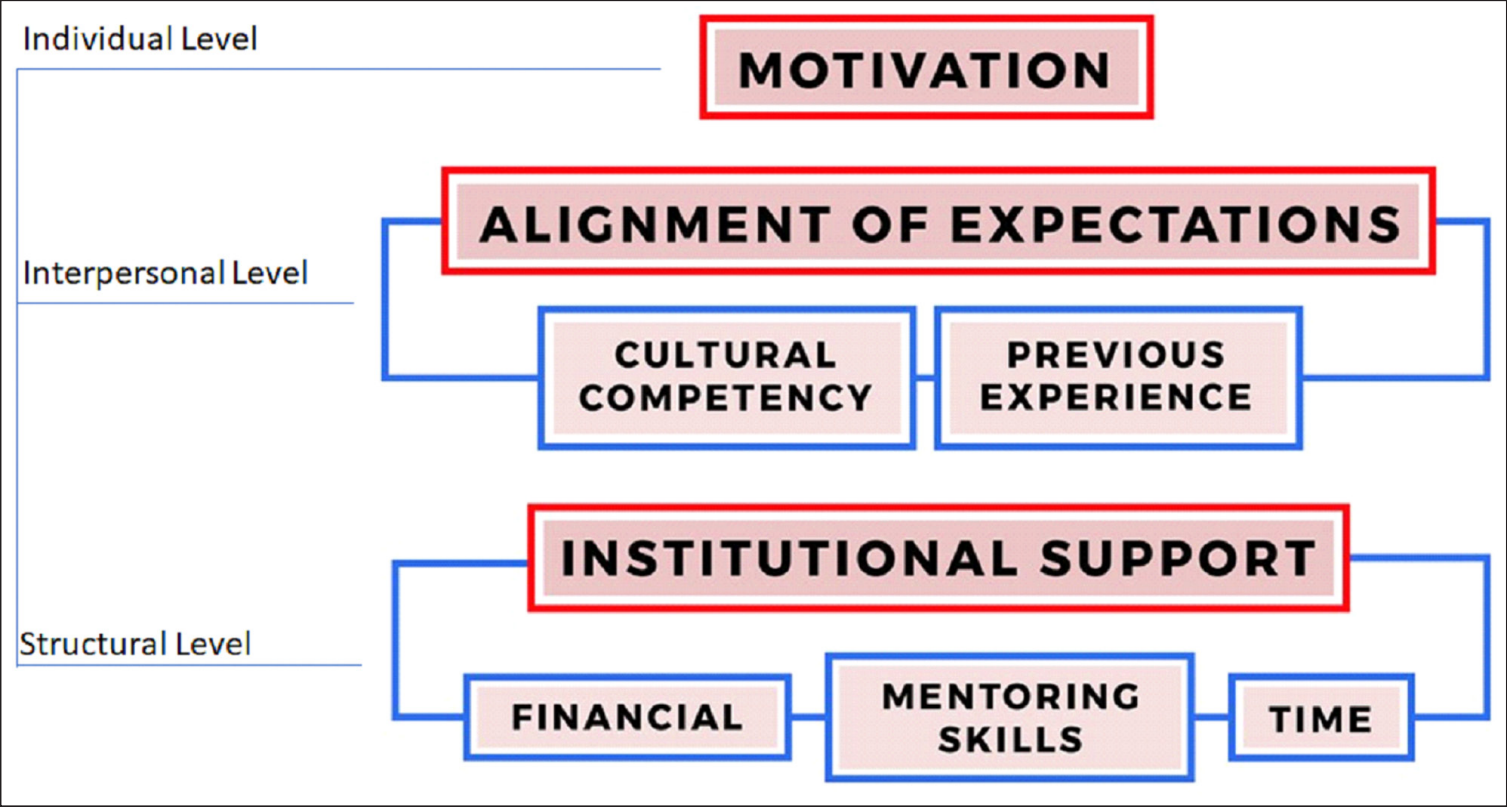
Building Blocks of Global Health Mentorship: A Socio-Ecological Model.

**Data Analysis.** Transcripts were line-by-line inductively coded using a combination of Microsoft Word and Atlas. TI. The study team developed a preliminary codebook of open codes by emerging theme, which were later organized in sets by axial codes drawing from corresponding levels of our SE model. Based on the emerging themes in the analysis, we built the theoretical model presented in Figure 1 that best explains the data.

## Results

Multiple themes were identified at different SE levels as influencers of the global health mentoring experience. Table 1 shows the key findings mapped to the Building Blocks of Global Health Mentorship Model. These findings are described in more detail below.

**Table 1:** Key Findings Mapped to the Building Blocks of Global Health Mentorship Model.

Mentorship Model Level	Key Results	Illustrative Quotations

**Individual Level**		

Faculty Motivation	– Faculty gain personal and professional satisfaction from sharing experiences. – Faculty enjoy preparing future practitioners and leaders. – Students provide needed assistance with projects.	I think oftentimes, my mentees inspire me as well because they look at things from a different perspective, and oftentimes, their perspective is a lot more fresh … they’re also very enthusiastic … [Faculty IDI_14]
Trainee Motivation	– International experiences help build skills in international settings and provide opportunity for immersion in new cultures.	I think at the end of the day it shows that you’re really adaptable and you’re independent. If you go somewhere that is completely new to you and you work with a completely new group of people, if you manage people or do whatever, I think it always looks good to any employer, whether you’re here or you’re international or wherever, that you can rise to challenges, you can be independent; you have to be a little brave to be able to do that and stand on your own. [Student_IDI_3]
**Interpersonal Level**		

Alignment of Expectations	– Increased academic costs have changed student expectations for mentorship. – Faculty seek students with previous work experience, cultural adaptability, and who are responsible. – Enumeration of expectations by all parties is important. – Changing timelines for projects are challenging to anticipate and require flexibility.	I think students oftentimes and/or trainees actually don’t know what they want. Or if they know, they don’t actually know how to verbalize it … and-and I think that’s actually the hardest battle, is really kind of knowing their expectations and being realistic with themselves, and being honest with their mentors. [Faculty_IDI_10]
Cultural Competency	– Student maturity and experience in low resource settings is a key consideration for faculty. – Cultural adaptation processes are important (e.g., learning the local language). – Faculty and students acknowledge unique learning and work environments in different countries.	There have been a couple of small challenges with some of the students being … whether unprepared or a little or maybe perhaps not mature enough to be travelling, and in a setting where it is not easy to get by if you don’t have a little bit of experience. That has taught me. That is why I have started to send people who are less experienced … with other people, this is true mostly for undergrads. [Faculty_IDI_12]
**Structural Level**		

Financial	– Protected time and financial support for mentorship are frequently lacking but may be catalysts for success. – Financial support for student health and wellness (e.g., vaccinations) is needed.	At the school, a common complaint from faculty is that you’re usually not compensated for advising and sometimes not for teaching and things like that, so they feel that if that’s an objective for the school, then faculty should have that time—you know, half a day or one hour, one to two hours or something like that, that they could focus on mentoring and advising and investing into students and having that time set because everybody travels and you’ve got research proposals to write and it’s just survival. [Faculty_FGD_2]
Time	– Some institutional work environments better promote mentorship and collaboration. – Faculty face pressure in academic settings to balance their success with their mentees’.	And students get disillusioned when they’re given an advisor and the advisor is never there. They knock on their doors, they’re traveling, they don’t respond as quickly, and so we need education with both making faculty sensitive and making students sensitive to this kind of academic environment, which is different from other universities. [Faculty_FGD_2]
Mentoring Skills	– Faculty are not formally trained in mentorship. – Support groups may help foster skills in less-seasoned mentors and provide spaces for open discussion and documentation of issues.	Look, like, no one took me to mentorship school, you know? [Faculty IDI 13] We’ve tried to institute a sort of advisor support group, and I’ve had about four of them this past year, and it’s a mix. There are some very senior advisors and some junior people, and we just sort of talk about best practices and what works, and I’m trying to kind of create … a sort of standard or an approach to improving the advising. [Faculty_FGD_1]

### Individual Level – Motivation

Faculty and students were asked to share their motivation and rationale for participating in student-faculty mentored overseas experiences. Many students and faculty described their motivation for engaging in global health training in terms of the educational and practical value of exposing and engaging students in overseas public health field work.

#### Faculty Motivation

Faculty expressed personal and professional satisfaction from sharing their experience and seeing students grow by witnessing the before and after connection of classroom learning with real situations.

Many faculty members acknowledged the link between teaching and mentoring to prepare future practitioners. Some faculty described student contributions to work on field projects as important to achieving the aims of the project itself. Other faculty described a desire to build and sustain their public health field of interest by preparing students to be leaders.

Some faculty were motivated by learning from and being inspired by students’ enthusiasm, especially when their interests align. One faculty said:

I think oftentimes, my mentees inspire me as well because they look at things from a different perspective and, oftentimes, their perspective is a lot more fresh … they’re also very enthusiastic, and it’s great because sometimes I feel like you get pretty inundated with the day-to-day and you’re like ‘OK same-old same-old,’ but from somebody who’s never seen this before, it’s not the same-old same-old, you know, so that really re-energizes you and inspires you. [Faculty IDI_14]

Personal mentoring experience (i.e., how they were mentored or not mentored) was an influence for some faculty.

#### Trainee Motivation

Students frequently described being motivated by the skills-building opportunity provided by field work. Students described wanting to build their careers and anticipated their experiences would give them the opportunity to apply classroom theory to real-world problems. One student reflected on the unique skills acquired while working overseas and their relevance to their future career, stating:

I think at the end of the day it shows that you’re really adaptable and you’re independent. If you go somewhere that is completely new to you and you work with a completely new group of people, if you manage people or do whatever, I think it always looks good to any employer, whether you’re here or you’re international or wherever, that you can rise to challenges, you can be independent; you have to be a little brave to be able to do that and stand on your own. [Student_IDI_3]

Some chose a faculty’s project based on a desired location or country, while others described a desire to immerse themselves in any new culture. When asked in routine post-return program evaluations why they chose to complete an overseas experience, almost half indicated that it was a requirement for a degree, and two-thirds stated that they were considering a career in global health.

### Interpersonal Level – Alignment of Expectations

Faculty and students discussed the expectations they had of each other and how to manage them. One faculty commented on the role of increased academic costs and their influence on student expectations for mentorship:

As tuition goes up, students have greater expectations for employment. When there was low tuition, people went to university to become a better person, but if they take out huge loan payments, they expect the training, they need the skills, they need the supervised experiences, they need to be on the ground collecting data, gaining marketable skills, and that is all linked to mentoring. [Faculty_IDI_11]

Several faculty described student qualities they deemed important for a successful mentorship in global health, including humility and cultural adaptability, a sense of adventure, and responsibility. When speaking about what they look for in mentees, one faculty said they look for someone:

Who is outgoing, who has traveled before, who has traveled in a foreign country is an advantage, but not a requirement, someone who is comfortable with a bit of uncertainty, and someone who is comfortable working and living in a cross-cultural setting is probably the most important one. [Faculty_IDI_1.1]

Previous work experience was mentioned by several faculty as a strong benefit to student success in the field. A few also described considering a student’s overall interests and goals when deciding what project to assign a student to. A few students described gaps between what they expected they would be doing or the level of support they thought they would receive and what they did in-country.

Both groups described having a series of conversations to set initial expectations, and one faculty member mentioned that they expected students to tell them what their limitations or skills were. Another faculty described the challenges associated with asking students to enumerate their expectations:

I think students oftentimes and/or trainees actually don’t know what they want. Or if they know, they don’t actually know how to verbalize it … and-and I think that’s actually the hardest battle, is really kind of knowing their expectations and being realistic with themselves and being honest with their mentors. [Faculty_IDI_10]

Several students and faculty commented about the need to accommodate changing project timelines. One student described their experience as different than originally envisioned:

I didn’t get a project in the sense that I was collecting data … It was more like program management type work, and that wasn’t what I was expecting originally.

Another student who reported working abroad before noted that prior field experience helped them to be more realistic about their time overseas:

My expectations were that things would change once I got there … maybe if you’ve never gone abroad and this is your first time, you may feel that it’s a little disappointing [if] you expected that ‘this is what I’ll be doing,’ and then you get there and things are totally different. Whereas for me, I kind of went in with the expectations that things will probably change. [Student_IDI_3]

### Interpersonal Level – Cultural Competency

Faculty were asked to describe the process by which they selected mentees to serve at an overseas practicum site. The concept of a student’s maturity came up often in faculty interviews as an external marker of a student’s cultural competency. Several indicated that maturity is informed by prior experience in a low- or middle-income country (LMIC) and is a key difference between undergraduate and graduate students. One faculty said:

There have been a couple of small challenges with some of the students being … whether unprepared or a little or maybe perhaps not mature enough to be travelling, and in a setting where it is not easy to get by if you don’t have a little bit of experience. That has taught me. That is why I have started to send people who are less experienced … with other people, this is true mostly for undergrads. [Faculty_IDI_12]

Attempting to adapt to the local culture, such as learning the local language to communicate with all team members, is important to faculty. One faculty said students need to be aware of their actions and behaviors because the students can be viewed as extensions of the professor:

As an expatriate, you know, non-national, it can often be very obvious that you are … someone associated with X project, and your behavior at all times is being scrutinized by the local community with whom we continue to work even after the students [depart]. So that, that student’s, behavior, during and after office hours is continuously important. And, so for example, that kind of sensitization is critical. [Faculty_IDI_2.1]

Both students and faculty acknowledged that learning and work environments in different countries will be different from the United States and require adaptation.

### Structural Level – The Institution

#### Financial

Faculty identified protected time and financial support as catalysts to mentorship success but were also frequently lacking. One faculty noted:

At the school, a common complaint from faculty is that you’re usually not compensated for advising and sometimes not for teaching and things like that, so they feel that if that’s an objective for the school, then faculty should have that time—you know, half a day or one hour, one to two hours or something like that, that they could focus on mentoring and advising and investing into students and having that time set because everybody travels and you’ve got research proposals to write and it’s just survival. [Faculty_FGD_2]

Faculty identified critical needs of the students. They felt it is important to support the safety and health of students while traveling. One area of need is financial support for the cost of vaccinations relevant to the students’ destinations:

I mean the thing we sometimes struggle with are the vaccines and how to cover those because depending on what people’s previous vaccines are like some people are pretty up to date and don’t need much, but other people who need more, it can get quite expensive, but, we’re a public health school, so I sort of feel very strongly about [both laugh] people shouldn’t go there without being properly vaccinated because of money. [Faculty_IDI_4.1]

#### Time

Faculty discussed how some work environments and collaborative partnerships promote mentorship while others do not. A few faculty mentioned working with partners who see trainees as extensions of Hopkins and thus not their responsibility to train or mentor. One faculty reflected on the influence of the academic environment on mentoring:

And students get disillusioned when they’re given an advisor and the advisor is never there. They knock on their doors, they’re traveling, they don’t respond as quickly, and so we need education with both making faculty sensitive and making students sensitive to this kind of academic environment, which is different from other universities. [Faculty_FGD_2]

Several faculty members also described the tension between needing to respond to institutional measures of their own academic success and those that are more student-centered. Many felt that in academic environments success is traditionally measured in part by outputs produced (i.e., papers, goods, etc.), which could provide opportunities for collaboration between students, faculty, and collaborators, but also could take away from time for mentorship. Faculty members also noted that these traditional metrics may not capture more student-centered metrics of success, such as emotional intelligence. One faculty said:

So when we look at the performance metrics of the school, we’re always focused on the cognitive intelligence and not much on social and emotional intelligence, which is what it takes to work with people at least overseas. [Faculty_FGD_2]

#### Mentoring Skills

Some faculty reflected on how they acquired mentoring skills and identified the need for additional mentorship training and education:

Look, like, no one took me to mentorship school, you know? [Faculty IDI 13]

I’ve learned … I think that people have differing levels of how much they want me to be involved. Some people want a lot closer involvement because they don’t feel quite as confident themselves doing things. Other people feel like if I’m getting too involved then I’m not allowing them to kind of find their own voice, like, their own style. [Faculty IDI 13]

One interviewee mentioned discussion groups, initiated by the institution, as a tool that has had some success in promoting good practices and supporting junior mentors:

And [CGH] always organizes a support group for all the faculty that’s mentoring students …. To share those feedback …. And it’s good to hear …. Whether it’s good or bad …. Especially the bad is good to hear as a reminder to fix it next time. [Faculty_IDI_6]

One faculty member described an ad hoc strategy for bringing together other faculty to discuss common issues in advising and mentoring students:

We’ve tried to institute a sort of advisor support group, and I’ve had about four of them this past year, and it’s a mix. There are some very senior advisors and some junior people, and we just sort of talk about best practices and what works, and I’m trying to kind of create … a sort of standard or an approach to improving the advising. [Faculty_FGD_1]

While not focused specifically on global health experiences, such groups might provide a forum for open discussion and documentation of key issues and strategies for learning and improving best practices for mentoring in academic settings.

## Discussion

The study highlighted several factors that contribute to effective mentorship during overseas training experiences, which are best represented by our theory-building model in Figure 1, the most salient of which are motivation, alignment of expectations, cultural competency, and institutional support.

Faculty and student motivation have overlapping themes. Both see mentorship relationships as part of their respective roles in an academic program. Students are motivated to apply skills in the real world, which corresponds to the faculty motivation to have students make meaningful contributions to their field projects. Faculty were more likely to report personal and professional satisfaction with the mentoring relationship. Alignment of expectations is a main factor for both groups; faculty spoke about students’ realistic reflection, preparation, and communication of expectations being a factor, while students reported disappointment when expectations were misaligned.

Cultural competency was acknowledged by both groups as important for navigating the global health environment and for interpersonal relationships. The role of the academic institution emerged as one of the most salient influencers on the faculty member’s capacity to serve as a mentor. Some faculty felt underprepared and overwhelmed by institutional and student expectations. Both faculty and students described logistical and emotional challenges of overseas experiences, which influenced the mentorship relationship.

Mentorship has long been recognized as a supervisory tool to improve satisfaction and work quality [16]. Global health work is often at the local community level, requiring skills and modeling of cultural competency. Providing support to global health mentors is critical to expanding the available pool of future mentors, providing training opportunities for mentees, and improving relationships with international colleagues and local communities. Multiple studies have recommended the development of formal policies, programs, and structures, including, but not limited to, mentorship training, peer support, and monetary support [17,18,19,20]. Mentors who participate in training programs have reported increased skills in establishing expectations with mentees and self-reported improvement in mentoring skills [21]. In 2008, Keyser et al. provided a framework for institutions to promote research mentorship and argued research mentorship “cannot be left to chance” [19]. In global settings, where challenges experienced by mentors and mentees can be even more acute, institutional support of mentorship cannot be overstated. Investigators at Makerere University College of Health Sciences (MAKCHS), Uganda, conducted a survey of mentors and mentees and similarly concluded that institutional support is critical to for a successful training in global health [22]. Agencies such as the World Health Organization, UNICEF, the Bill and Melinda Gates Foundation, the Centers for Disease Control and Prevention, and Save the Children are global health organizations that seek qualified professionals with field experience in a low resource setting. The incorporation of a mentorship plan that addresses the individual, interpersonal, and structural levels as part of a global field site curriculum provides students a real-world experience that helps them transition to organizations such as these upon graduation.

This study was limited in its scope in that it originated as a program evaluation within the context of a student project within a qualitative methods class and was continued by the authors over subsequent years. Over this period, themes emerged that informed later iterations, so in keeping with the reflexive and evolving nature of qualitative methods, earlier respondents were not asked questions posed to later respondents. Due to time restrictions imposed by the class format, the sample was small and selected by convenience. Both faculty and students were interviewed to capture a comprehensive perspective of the mentorship process. To minimize social courtesy bias from student respondents, all interviews were conducted by trained student interviewers. While this arrangement could influence the depth of probing or information provided during faculty interviews, the length and depth of faculty responses do not suggest this occurred.

The model that we have developed communicates factors critical to the success of mentor-mentee relationships and can be used to guide planning for successful student experiences at global field sites. Our research questions are focused on global health training, but many of these factors are essential to strong mentorship across multiple disciplines [16]. While our interviews are limited to our university community, from informal conversations with colleagues across varied institution types and geographic locations, we recognize that many factors articulated in the model are common challenges faced by those engaging in global health. A next phase of this research includes interviewing overseas colleagues on their thoughts and practices regarding mentorship.

Based on the findings presented in this study, we present the following recommendations to enhance global health mentorship, particularly for international electives.

### Recommendation #1

Acknowledge that motivation can impact mentorship. Encourage dialogue between mentors and mentees about motivation to encourage both parties to think beyond the project aims and to provide a platform for building a mentorship relationship. Being aware of what motivates each party can help both students and faculty find commonalities and craft experiences that help satisfy each party’s personal motivations and interests.

### Recommendation #2

Describe expectations beyond technical skills. Faculty mentor and student interviews cited misalignment of expectations as a source of conflict. In reviews of CGH programming, we found that these conflicts typically fall into two categories: technical, relating to project-specific knowledge and responsibilities, and daily living, relating to a wide range of issues encompassing mentorship structure between international and local staff, level of communication, culture shock, living arrangements, and previous international experience, among others.

In the results presented in this paper, the latter category comprises the source of the majority of reported conflicts due to misaligned expectations. In response, the CGH now actively requires mentors and students to document their individual communication plans and to agree to engage in discussions around the field site, cultural context, local collaborators, and so forth as a mechanism for avoiding potential misalignment and conflict. Faculty mentors should also recognize that they may not always be the most appropriate individual for these conversations, especially those regarding local conditions or other daily living considerations. In these instances, we encourage mentors to link students to previous student researchers or local collaborators.

### Recommendation #3

Recognize the institution as a fourth partner in the student overseas experience. Institutional curriculum requirements and policies can have an impact on student and faculty abilities to engage in effective mentorship and to conduct research and practice in international settings. For example, at the authors’ university, to address and standardize the mentors concerns, the annual seminar on student travel preparation was developed into an online safety and travel preparedness course offered several times a year and is now mandatory for all students in the Department of International Health and all students participating in overseas experiences through the Center for Global Health.

### Recommendation #4

Institutions (i.e., organizations that offer overseas experiences) should provide protected faculty time and training on mentorship. Throughout the interviews, many faculty described mentorship as an abstract concept they felt they were expected to understand and implement with minimal support and oversight from the institution. Comprehensive mentorship training that addresses the challenges faculty described above, particularly from professional development specialists within or external to the institution, could mitigate some of the frustrations encountered between faculty and students. Purposeful building of a mentorship community allows for a tangible space in which mentors can share their experiences, reflect, and learn from peers. Development of a core mentorship skills checklist may also provide a more systematic way of teaching and evaluating mentorship. Protected time to mentor may alleviate stress for faculty who seek their own funding and could allow faculty to spend more time engaging with mentees.

### Recommendation #5

Institutions should designate funding mechanisms to support costs of travel and associated fees that are typically borne by students and, occasionally, by faculty. Consistently in the authors’ post-return programmatic evaluations, students indicate they would not have been able to complete the experience without external funding. Many faculty would also be unable to mentor students without external funding sources to support travel and costs of living. Institutional funding mechanisms would provide more opportunities for students and faculty to work together for longer-term experiences.

These recommendations are focused on the faculty-student dyad, but local partners at global field sites remain an essential component of that relationship and experience. Increasingly, conversations in global health are centered on how to build mutually beneficial partnerships. The Center for Global Health and the Department of International Health are now building on ways to best support and implement these recommendations in programming and among our faculty and students.

## Conclusion

Many factors, ranging from individual to institutional, influence global health mentorship for both mentors and mentees, which in turn influence international experiences. The underlying role of institutional support emerged as a highly salient influencing factor and serves as the base of the building blocks model. Global health training programs should harness and nurture the faculty and students’ motivation and expectations, as well as provide improved support to mentors while increasing their capacity to financially support students working in global health settings.

## Data Accessibility Statement

All authors had access to the data and played a role in conceptualizing and authoring the manuscript.
